# The Metric System

**Published:** 1889-06

**Authors:** J. F. Evans

**Affiliations:** Wellborn, Texas


### THE METRIC SYSTEM.

By J. F. Evans, M. D., Wellborn, Texas.

[For Daniel's Texas Medical Journal.]

THIS little article may be of some advantage to the readers of the Journal.

I have always had an idea that the metric system, which is now being so extensively used by some of our home journals and by writers on medicine generally, was impossible to comprehend. So disgustful was it, that when I came across a formula given in the metric system, I passed it by with a mingled feeling of disgust and respect; disgust because of my inability to profit by its teachings, and respect because of its supposed utter incomprehensibility. A half hour's study will make it plain to the medical brethren, for it is the easiest system of all.

We all know the unit of weight of the metric system is the "gramme." Now, to obtain fractional parts prefix the Latin word "deci" for tenths, "centi" for hundredths, and "milli" for thousandths. Written decimally one decigramme is .1, one centigramme .01, one milligarmme .001.

In order to obtain higher quantities prefix the Greek word "deka," or ten times, "hecto," or one hundred times, "kilo,"

or one thousand times, and then we read "decagramme," or ten grammes, "hectogramme," or one hundred grammes, "kilogramme," or one thousand grammes.

TABLE OF COMPARISON.

| Fractional. | | Unit. | |
|---|---|---|---|
| 1 Decigramme, | .1 | 1 Dekagramme, | 10. |
| 1 Centigramme, | .01 | 1 Hectogramme, | 100. |
| 1 Milligramme, | .001 | 1 Kilogramme, | 1000. |

In order to obtain equivalents in troy or apothecaries weights —one gramme is equal to about 15½ grains, or, to be accurate, it is equal to 15.432 grains, consequently one decigramme (.1) is one-tenth of 15.432, which is 1.5432; therefore we might say one decigramme is about one and a half grains. Now, to obtain fractional parts of the gramme move the decimal point one place to the left for tenths, two places for hundredths and three places for thousandths, and to obtain the multiple move the decimal point as above, but to the right instead of the left. The following table gives the equivalents:

| | | |
|---|---|---|
| 1 Milligramme, | .0154 | grains. |
| 1 Centigramme, | .1543 | " |
| 1 Decigramme, | 1.5432 | " |
| 1 Gramme, | 15.4323 | " |
| 1 Dekagramme, | 154.323 | " |
| 1 Hectogramme, | 1543.23 | " |
| 1 Kilogramme, | 15432.3 | " |

The table of measure is constructed on the same principle; the unit of measure is the litre, which is nearly a quart. It is too large a unit for our practical purposes, and the cubic centimetre, which is the volume of a gramme of water, is the unit used. A litre contains 1000 cubic centimetres. There are about 30 cubic centimetres in a fluid ounce, or, to be accurate, 29.57 cubic centimetres in an ounce. Then the cubic centimetre is equal to 1 gramme, in weight 15.432, which is a gramme.

The advantage of this system is this: Suppose you wish to make a two per cent solution of cocoaine. Now, that means that you are to dissolve 2 grs. in 100 grs. of water. Of course the doctor has no facilities for weighing liquids, consequently he must reduce it to minims, or parts of a fluid ounce, which will require quite a calculation. Now mark the simplicity of the metric system: To make a two per cent. solution of cocoaine we just dissolve 2 grammes in 100 grammes of water. Now, as a gramme of water is a cubic centimetre, instead of weighing the water or going through a tedious arithmetical calculation, all we have to do is to substitute cubic centimetre for gramme. To sum up the whole, 1 gramme equals a little over 15 grains, 1 dekagramme about 1½ grains, 1 centigramme about 1-7 of of grain, 1 milligramme about 1-64 of a grain. A fluid ounce is equal to about 30 cubic centimetres, a teaspoonful is equal to about 4 cubic centimetres. The metre, the unit of length, is 4 inches longer than our common yard. An inch is equal to about two and a half centimetres or twenty-five millimetres.

In conclusion, the metric system is being so generally used, almost an imperative demand is made for its adoption and comprehension by the medical men who went out long before it was thought of by the medical authors who are now using it to such an advantage.

Still I don't like it.

